# Magnetic Resonance Imaging for Characterization of a Chick Embryo Model of Cancer Cell Metastases

**DOI:** 10.1177/1536012118809585

**Published:** 2018-11-05

**Authors:** Anne Herrmann, Arthur Taylor, Patricia Murray, Harish Poptani, Violaine Sée

**Affiliations:** 1 Department of Biochemistry, University of Liverpool, Liverpool, United Kingdom; 2 Centre for Preclinical Imaging, Department of Cellular and Molecular Physiology, University of Liverpool, Liverpool, United Kingdom

**Keywords:** MRI, chick embryo, imaging metastasis, neuroblastoma, *in ovo* imaging

## Abstract

Metastasis is the most common cause of death for patients with cancer. To fully understand the steps involved in metastatic dissemination, *in vivo* models are required, of which murine ones are the most common. Therefore, preclinical imaging methods such as magnetic resonance imaging (MRI) have mainly been developed for small mammals and their potential to monitor cancer growth and metastasis in nonmammalian models is not fully harnessed. We have here used MRI to measure primary neuroblastoma tumor size and metastasis in a chick embryo model. We compared its sensitivity and accuracy to end-point fluorescence detection upon dissection. Human neuroblastoma cells labeled with green fluorescent protein (GFP) and micron-sized iron particles were implanted on the extraembryonic chorioallantoic membrane of the chick at E7. T_2_ RARE, T_2_-weighted fast low angle shot (FLASH) as well as time-of-flight MR angiography imaging were applied at E14. Micron-sized iron particle labeling of neuroblastoma cells allowed *in ovo* observation of the primary tumor and tumor volume measurement noninvasively. Moreover, T_2_ weighted and FLASH imaging permitted the detection of small metastatic deposits in the chick embryo, thereby reinforcing the potential of this convenient, 3R compliant, *in vivo* model for cancer research.

## Background

Metastasis accounts for 90% of cancer deaths,^1^ yet it is one of the most poorly understood aspects of tumor progression. In order to reduce metastasis-associated mortality, it is crucial to understand how, when and where metastasis occurs. However, small size, heterogeneity, and large dispersal of disseminated cancer cells, combined with the limited sensitivity and spatial resolution of current clinical imaging methods, make their early and reliable detection challenging. Metastatic dissemination is a complex process involving several steps from the initial detachment of cells from the primary tumor, diffusion within the surrounding stromal tissue, degradation of the extracellular matrix, and intravasation into the blood stream. Once in the circulatory system, tumor cells not only have to survive the hostile environment, but also attach to the endothelial cells of the vessel wall, extravasate in the extravascular tissue, and proliferate in the metastatic site to form secondary tumors.^2^ Although many of these steps have been studied at a molecular level *in vitro*, visualization of the dynamic events *in vivo* remain elusive.

Currently used methods to detect the presence of metastasis *in vivo* in experimental studies rely mostly on end-point measurements and require the termination of the experiment and organ dissection. Modern imaging modalities such as magnetic resonance imaging (MRI), positron emission tomography or bioluminescence imaging allow non-invasive and longitudinal imaging of metastatic dissemination in whole organisms. In addition, MRI provides enhanced soft tissue contrast, 3-dimensional (3-D) anatomical information and high spatial resolution. Although the detection of primary tumors with MRI is already a routine practice, finding metastasis is more challenging as the metastatic cell population is heterogeneous and usually consists of single cells or a small group of malignant cells present in various tissue types, which makes their detection difficult. The use of contrast agents like iron oxide nanoparticles or gadolinum-based agents for cell labeling can enhance contrast and thus detection limit. Iron oxide particles cause a distortion in the magnetic field leading to a change in T_2_/T_2_* relaxation and are mainly used to generate hypointense contrast on MRI.^3,4^ Although a broad range of iron oxide particles are available for cell tracking, micron-sized iron particles (MPIOs) are of special importance as they are not only taken up efficiently and rapidly by cells but also enable prolonged imaging due to their ability to label cells with a single particle only.^5-7^ Using contrast agents, metastasizing cells could be detected in the lymph nodes,^3,8-10^ liver,^11-13^ and brain^14^ of rodents. Foster et al. reported the detection of approximately 100 MPIO-labelled cells after direct implantation of melanoma cells in the lymph node.^3^ Even detection at single cell level was observed as small metastatic deposits could be found in livers postmortem^12^ and in the brain after injection into the left ventricle of the heart.^14^

While rodents constitute the most widely used preclinical model for studying tumor development and metastasis, the chick embryo is a versatile 3R compliant model that is readily accessible *in* or *ex ovo*, nutritionally self-sufficient, cost-efficient, and phylogenetically more similar to mammals than several other models of replacement, such as the zebrafish or nematode worm. The main advantage, when models for tumor formation are considered, is the accessibility of its chorioallantoic membrane (CAM), a highly vascularized extraembryonic membrane that is located directly beneath the eggshell. Thus, tumor cells can be engrafted easily, noninvasively and in the absence of an “interfering” immune system, since the chick embryo is immunodeficient at earlier stages of development, when cells are implanted. Within days, tumor formation occurs and, in the case of aggressive tumors, metastasizing cells can colonize the host’s organs via hematogenous metastasis.^15^ However, despite all the advantages of the chick embryo, its potential has not been fully exploited so far. One reason for this might be the difference in protocols required for the successful MRI without motion artefacts. While the topical application of anaesthetic agents can be used to achieve motionless imaging, it can impair the embryo’s survival and thus render this method impractical for longitudinal imaging. Cooling the embryo on the other hand has been successfully used for repeated MRI at different embryonic stages.^16,17^ Together, it makes the chick embryo model an attractive alternative for *in vivo* animal experiments.

We evaluated the advantages and limitations of MRI to study metastatic dissemination of neuroblastoma in the chick embryo, as a preclinical model. We have shown previously that we can induce metastasis *in vivo* by preculturing neuroblastoma cells in hypoxia or by treating with the hypoxia mimetic drug dimethyloxalylglycine, where cells metastasize in 52% and 75% of cases, respectively.^15^ While MRI has previously been used to monitor tumor growth in the chick embryo,^17^ we here investigated the feasibility of MRI to detect metastatic deposits of MPIO-labelled neuroblastoma cells in the chick embryo.

## Methods

### Cell Culture

The human NB line SK-N-AS (ECACC No. 94092302, Salisbury, UK) was grown in minimal essential medium supplemented with 10% (v/v) fetal calf serum and 1% (v/v) nonessential amino acids (both Life Technologies, Carlsbad, California) and maintained in a humidified incubator at 37°C, 5% CO_2_. For hypoxic studies, cells were maintained at 37°C, 5% CO_2_, and 1% O_2_ (Don Whitley Scientific—Hypoxystation-H35, Bingley, UK).

### Stable Cell Line Generation and Cell Labeling

Lentiviral particles were produced with the transfer vector pLNT-SFFV-EGFP^18^ as described previously.^6^ For cell labeling, 2 × 10^6^ SK-N-AS cells were seeded in a T-75 flask and allowed to grow for 24 hours. Then 20 µM of Suncoast Yellow Encapsulated Magnetic Polymers (Bangs Beads, Stratech Scientific, Suffolk, England) were added directly to the complete culture medium and cells were allowed to grow for further 48 hours. After the labeling period, the cells were carefully washed with phosphate buffered saline (PBS) to remove excess contrast agent, harvested and used for *in vivo* studies. Stability of the bang beads over time and their remaining numbers in cells upon multiple cycles of cell division were previously tested.^6^

### Primary Tumor, Experimental, and Spontaneous Metastasis Assay

For the observation of the primary tumor, CAM implantation at E7 was performed as described previously.^19^ In brief, fluorescent (GFP) and MPIO-labeled SK-N-AS cells were harvested and 1 × 10^6^ cells/µL were resuspended in serum free media. Chorioallantoic membrane implantation was achieved by transferring 2 µL of the cell suspension into the CAM membrane fold created by careful laceration of white leghorn chicken embryos.

For the observation of cells directly injected in the chick organs, fluorescent and MPIO-labeled SK-N-AS cells were harvested and 1 × 10^5^ cells/µL resuspended in serum free media and 0.15% (v/v) fast green (Sigma-Aldrich, Dorset, UK). Cell implantation was achieved by injecting 3 µL of the cell suspension into the brain of white Leghorn chicken embryos *in ovo* at E7 using a micro-capillary pipette.

For the observation of spontaneous metastasis, CAM implantation at E7 was performed as described above for primary tumor formation using hypoxic preconditioned neuroblastoma cells. In brief, fluorescent (GFP)-labeled SK-N-AS cells were preconditioned in 1% O_2_ for 3 days. Micron-sized iron particle labeling took place 48 hours prior harvesting. Cells were harvested and 1 × 10^6^ cells/µL were resuspended in serum free media. Chorioallantoic membrane implantation was achieved by transferring 2 to 10 µl of the cell suspension into the CAM membrane as explained above.

After cell implantation, eggs were maintained at 37°C and 40% humidity (Ovo Easy 380, Brinsea, Weston Super Mare, UK) until E11 or E14 and all animal work followed UK regulations (Consolidated version of ASPA 1986). For MRI scanning, embryos were removed from the incubator at E11 or E14, cooled at 4°C for 60 minutes and then imaged. The cooling protocol was previously described by Zuo et al., however, here the same cooling duration was used for both E11 and E14, as it was enough, in our room temperature conditions, to avoid chick movement and to keep the egg cold upon imaging. In the case of time-of-flight angiographic MRI (TOF MRA), embryos were not cooled but anaesthetized with 3.6 mM ketamine in 500 µL of PBS (Sigma-Aldrich) dropped directly onto the CAM prior to MRI. The protocol for ketamine application was optimized using different concentrations of ketamine (data not shown). The application of 3.6 mM ketamine in 500 µL of PBS resulted in MRI that was free of motion artefacts for 30 minutes (ToF scanning time was 12 minutes). While the embryos recovered well after anaesthesia, we cannot exclude an impact on long-term survival as mentioned by Zuo et al.^17^

### Fluorescent Detection of Tumor and Metastatic Deposits

Following MRI, a standard fluorescent stereo microscope (Leica M165-FC, Wetzlar, Germany) was used to image primary tumors and metastatic deposits. Tumors were removed from the CAM and were imaged from 3 different perspectives (dorsal, ventral, and lateral). Following removal of primary tumors from the CAM, embryos were dissected. Organs were removed and tumor cells and/or metastatic deposits identified by fluorescence.

Subsequently, tumor and organ samples were fixed for up to 12 hours in 4% formaldehyde for the preparation of 10 µm thick frozen sections. Frozen tissue slices were stained with Hoechst and analyzed with an epi-fluorescent microscope (Axio ObserverZ1, Zeiss, Oberkochen, Germany). A representative sagittal MRI slice was correlated with the section of the region of tumor or metastatic deposit.

### Tumor Volume Calculation

#### By microscopy

Excised tumors were imaged from 3 different perspectives (dorsal, ventral, and lateral). Average tumor volume was calculated as previously described^15^ using *V* = 4/3 × *π × l × h × d*, where *l* is length, *h* is height, and *d* is depth. The volume of tumors extracted from 8 chick embryos was analyzed.

#### By MRI

T_2_ weighted (T_2_W) images were used for tumor volume calculation. The tumor area was measured with ImageJ 1.48 (Wayne Rasband) in each slice and tumor volume was calculated using $V=\left(t+h\right)\sum_{i=1}^{N}A_{i}$, where *N* is the number of slices, *A_i_* the area of the region of interest (ROI) encompassing the tumor, *h* the slice gap, and *t* the slice thickness.^20^ The volume of tumors extracted from 8 chick embryos was analyzed.

#### In ovo MRI

**Figure 1.**
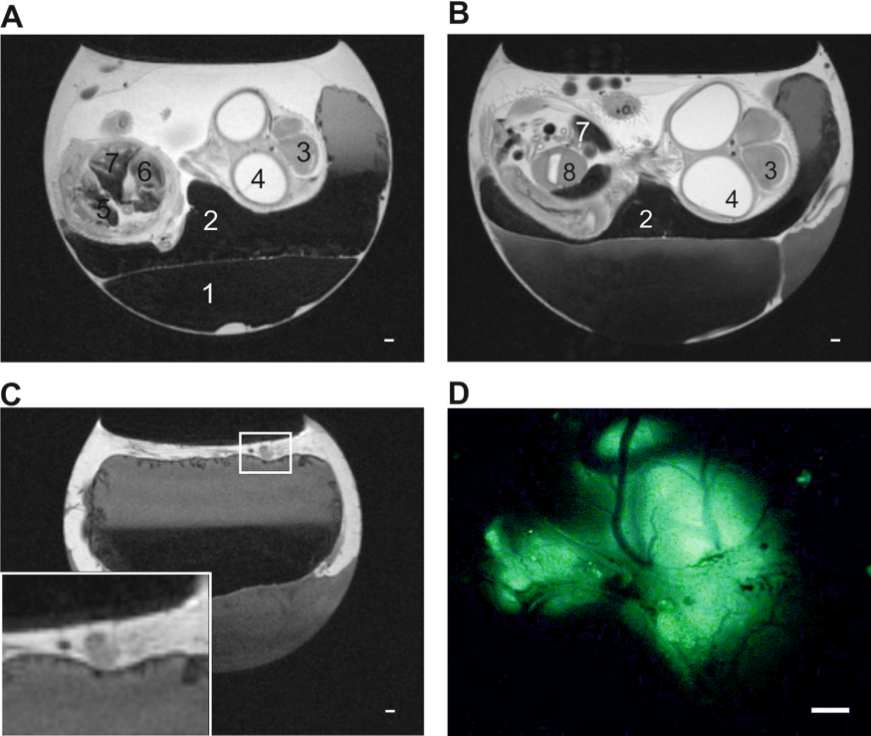
T_2_W images of tumors growing on the CAM (A and B) Representative Sagittal T_2_W MRI images of E11 (A) and E14 (B) chick embryo *in ovo*. Egg compartments like albumen (1), yolk (2) as well as chick embryo organs like brain (3), eyes (4), kidneys (5) heart (6) liver (7), and gizzard (8) can be identified. (C) Representative sagittal T_2_W MRI images of embryonated chicken egg at E14 *in ovo*. Extraembryonic tumor can be identified on top of the CAM (zoom in inset) and correlates with fluorescent image (D). Due to the anatomy of the egg the primary tumor is not always located above the chick embryo and thus the chick embryo does not always appear in the same sagittal slice as the one showing the primary tumor. (D) The same tumor than in (C) was imaged with fluorescence microscopy. The picture is a representative image of the tumor formed by GFP-expressing neuroblastoma cells. Scale bars represent 1000 µM.

Magnetic resonance imaging data were acquired with a Bruker Avance III spectrometer interfaced to a 9.4T magnet (Bruker Biospec 90/20 USR, Billerica, Massachusetts) using a 74-cm transmit-receive resonator coil. Sagittal images of the chick embryos were acquired using following sequences: (1) high resolution TurboRARE T_2_ weighted (T_2_W) images with the following parameters: field of view 45 mm × 35 mm, matrix size 512 × 398 (256 × 198 for Figure 1A-C), slice thickness 0.4 mm (0.5 mm for Figure 1A-C), slice gap 0.3 mm, effective TE 35 ms, TR 7822 ms (6703 ms and 7262 ms for Figure 1A and Figure 1B/C, respectively), averages 5, slices 70 (60 and 65 for Figure 1A and Figure 1B/C, respectively), scan time 31 min 56 s (13 min 24 s and 14 min 31 s for Figure 1A and Figure 1B/C, respectively); (2) T_2_* weighted (T_2_*W) images using a fast low angle shot (FLASH) sequence with the following parameters: field of view 45 × 35 mm, matrix size 512 × 398, slice thickness 0.4 mm, slice gap 0.3 mm, effective TE 6.88 ms, TR 1135 ms, averages 3, flip angle 30°, slices 70, scan time 22 min 35 s; (3) angiography using a ToF sequence with following parameters: field of view 45 mm × 35 mm, matrix size 512 × 398, slice thickness 0.4 mm, slice gap 0.3 mm, effective TE 3.1 ms, TR 13 ms, averages 2, flip angle 80°, slices 70, scan time 12 min 4 s.

## Results

### T_2_W Imaging of Chick Embryos Allows Observation of Tumorigenesis and Embryonic Development

Fluorescently labeled (GFP) neuroblastoma cells were implanted on the CAM at E7 and tumor formation was assessed by MRI. Representative images from T_2_W multislice MRI scans obtained at E11 and E14 are shown in Figure 1A and B, respectively. Allantois, yolk sack, and chick embryo organs such as liver, kidneys, and heart can be clearly identified and studied over time. The cooling of the embryos at 4°C for 60 minutes prior to imaging reduced their movement for up to 60 minutes allowing motionless imaging. Tumors grown on the CAM can be easily identified by MRI (Figure 1C). Primary tumor dissection with a fluorescent microscope revealed that location and morphology of the tumor are in good correlation with the images acquired by MRI (Figure 1D).

### Micron-Sized Iron Particle Labeling Facilitates Tumorigenesis Observation and Allows Tumor Volume Measurement

**Figure 2.**
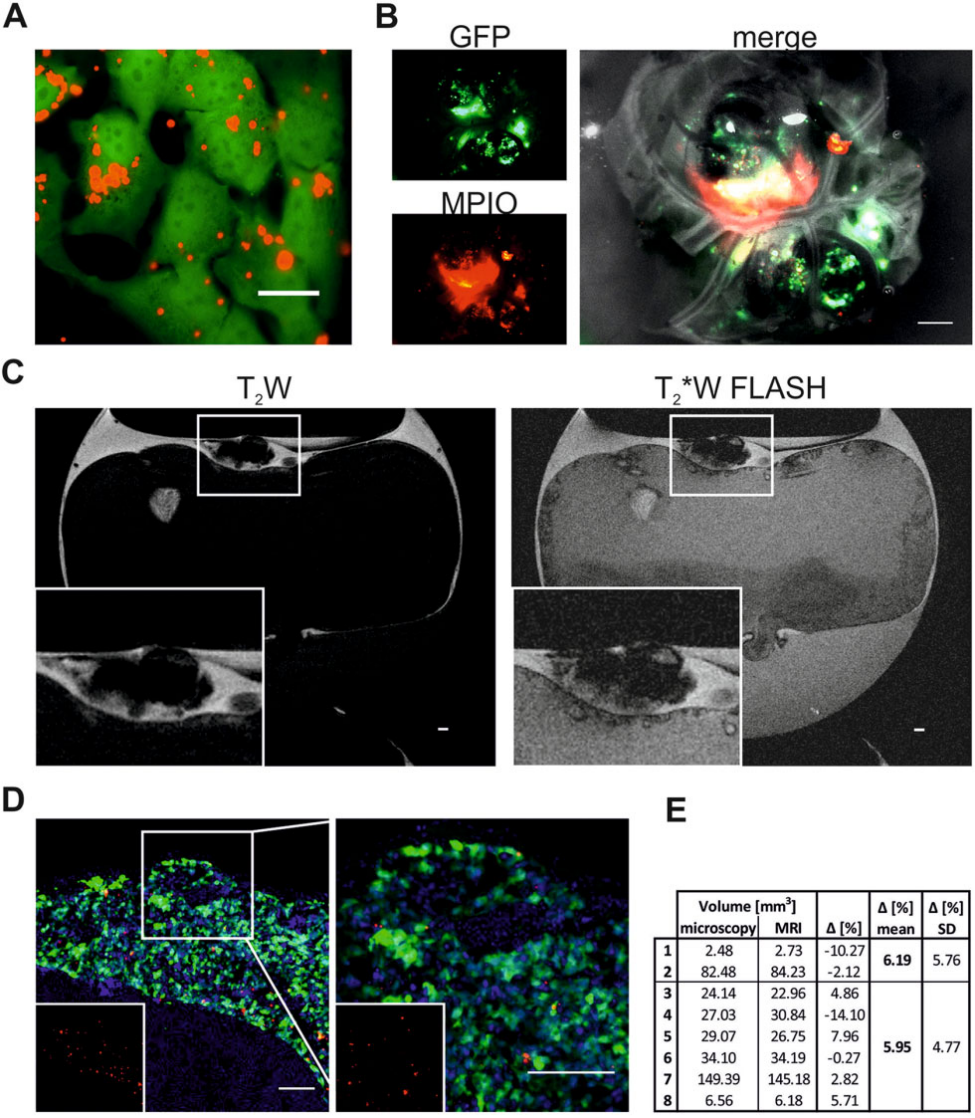
T_2_W and T_2_*W FLASH images of tumors labeled with MPIO (A) GFP-expressing SK-N-AS cells (green) 24 hour-postlabeling with 20 µM MPIO (Suncoast Yellow Encapsulated Magnetic Polymers—Bangs Beads, Red). Scale bar is 20 µm. (B) Single channel and overlay image of neuroblastoma tumor postdissection formed by GFP-expressing SK-N-AS cells (green) which were labeled with MPIO (red) 48 hours prior CAM implantation. Scale bar is 1000 µm. (C) Representative sagittal T_2_W and T_2_*W FLASH MRI images of embryonated chicken egg at E14 (a). Tumor formed by cells labeled with MPIO can be identified on top of the CAM (zoom in inset). Scale bar is 1000 µm. D, Representative image of tumor formed on the CAM by GFP-expressing SK-N-AS cells (green) labeled with MPIO (red). Nuclei are stained with Hoechst (blue). Inset shows MPIO only (red). Right image is 2.5× zoom. Scale bar is 100 µm. E, Comparison of tumor volume (mm^3^) measured by microscopy or MRI. Tumors 1 to 2 were formed by cells without MPIO, tumors 3 to 8 were formed by cells with MPIO. FLASH indicates fast low angle shot; MRI, magnetic resonance imaging; MPIO, micron-sized iron particles.

To investigate whether MPIO-labeling enhances the detection of primary tumors, GFP-expressing neuroblastoma cells were labeled with red fluorescent MPIOs for 48 hours prior to CAM implantation. Micron-sized iron particle uptake was efficient as all cells contained multiple MPIOs 24-hour postlabeling (Figure 2A). Micron-sized iron particle-labeled cells successfully formed tumors on the CAM and signal from GFP as well as MPIOs could be detected by fluorescence (Figure 2B). Tumor formation was then assessed by T_2_W and T_2_*W FLASH MRI scans. Using FLASH, areas containing cells labeled with MPIOs should experience an enhanced signal loss compared to other areas, such as blood vessels or tissue. Representative images from T_2_W and T_2_*W FLASH scans obtained at E14 show that, as with unlabeled cells, tumors could be readily identified in the MRI scans (Figure 2C). Tumors formed from MPIO-labelled cells, however, displayed a much stronger signal loss, which is expected given their iron oxide load. Primary tumor dissection revealed that location and morphology of the tumor were comparable to the images acquired by MRI (Figure 2B). Fluorescent images of frozen tumor sections revealed a homogenous distribution of MPIOs within the tumor (Figure 2D). Only a fraction of cells still contained MPIOs, which was expected due to extensive cell proliferation during tumor development *in vivo* and consequently, progressive dilution of the label between daughter cells. Micron-sized iron particles were only observed in GFP-labelled tumor cells and not in the surrounding chick tissue.

In order to determine whether MRI can also be used to determine tumor volume, tumor areas on sagittal T_2_W MRI slices displaying the primary tumor were measured and calculated as described in the methods section. Different amounts of cancer cells were implanted in order to compare a range of tumor sizes and observe whether the 2 methods relate for small as well as large tumors. In addition, we also compared labeled cells with unlabeled ones. Tumor volume estimates were then compared to those obtained from tumor excision and microscopy and were comparable with a difference of 6.19% and 5.95% for tumors formed by unlabeled and labeled cells, respectively (Figure 2E). The slight dissimilarity between the 2 methods can be explained by the difference in volume calculation. Although the volume measured in images obtained by microscopy assumes that the tumor is a spherical object, MRI allows a more precise estimation as the area of each slice displaying a tumor is considered. While it was easier to see the tumors when they were labeled with MPIO, it did not drastically change the ability to detect primary tumors and it had no impact on tumor volume measurements. Thus, MRI can easily be used to study the presence, progression, and volume of tumors noninvasively over time, in contrast to fluorescence microscopy, which necessitate tumor excision from the CAM.

### Micron-Sized Iron Particle Labeling Combined With T_2_W and FLASH Imaging Allows Detection of Metastasis

**Figure 3.**
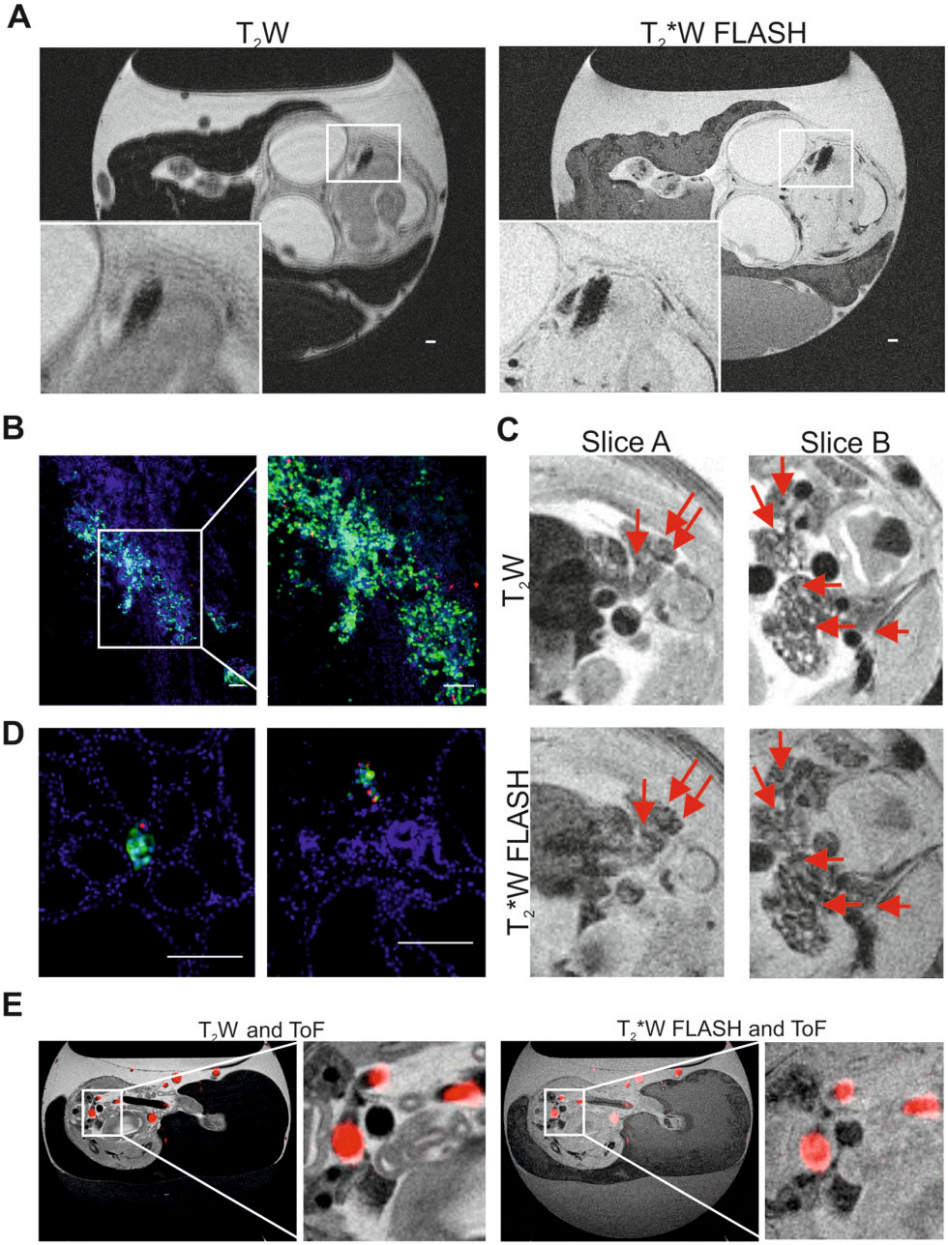
Magnetic resonance imaging of cell deposits and metastasis in the chick embryo organs (A) representative sagittal T_2_W and T_2_*W FLASH MRI of E14 chick embryo *in ovo*. Deposit formed by cells labeled with MPIO can be identified in the brain (zoom in inset) scale bar is 1000 µm. (B) Representative fluorescence microscopy image of brain slice showing cluster of GFP and MPIO-labelled neuroblastoma cells, and zoom. Scale bar is 100 µm. (C) Representative sagittal T_2_W and T_2_*W FLASH MRI of E14 chick embryo *in ovo*. Shown are two slices of the abdominal region and kidneys. Arrows indicate signal loss that intensified in the T_2_*W FLASH sequence and thus indicates the potential presence of metastasis. (D) Representative fluorescence microscopy image of kidney slices showing metastatic deposit of GFP and MPIO-labeled neuroblastoma cells. Scale bar is 100 µm. E, Representative sagittal T_2_W and T_2_*W FLASH MRI of E14 chick embryo *in ovo* overlayed with ToF MRA (red). FLASH indicates fast low angle shot; MPIO, micron-sized iron particles; ToF MRA, time-of-flight magnetic resonance angiography.

To first investigate whether MPIO labeling enables the detection of cells within the chick embryo organs, 3 × 10^5^ GFP-expressing and MPIO-labeled neuroblastoma cells were directly injected into the brain of the chick embryo at E7 and analyzed at E14. Representative images from T_2_W and T_2_*W FLASH scans obtained at E14 are shown in Figure 3A. A small region (2 mm × 1 mm) of signal loss can be observed in the brain indicating the presence of MPIO-labeled tumor cells. Size, shape, and location of the cell cluster correlate well with the fluorescent signal obtained by subsequent fluorescence microscopy and tissue analysis (Figure 3B). Like in the primary tumor growing on the CAM, MPIOs were homogenously distributed among the cell population, with a great proportion of the cells not containing MPIOs anymore.

We further evaluated whether MPIO labeling enables the detection of spontaneous and smaller metastasis using a spontaneous metastasis model in the chick embryo.^15^ We have previously shown that we can control metastasis of neuroblastoma cells by hypoxic preconditioning.^15^ However cells grown in normoxia are capable of tumorigenesis but not of metastatic invasion, cells grown in hypoxia (3 days in 1% O_2_) metastasize in 52% of the cases from the primary tumor into the chick embryo organs. While such metastatic phenotype was observed as an end point measurement upon chick organ dissection, the detection of metastasis in the chick embryo using imaging modalities has not yet been reported. The GFP-expressing and MPIO-labeled neuroblastoma cells were cultured under hypoxia, implanted on the CAM at E7 and their metastasis into chick tissues was assessed at E14, using T_2_W and T_2_*W FLASH scans (Figure 3C). Fast low angle shot MRI was applied in order to distinguish the regions of signal loss caused by small blood vessels, hemorrhagic areas, air-tissue interfaces such as the pancreas or areas devoid of proton signal such as the lungs from potential neuroblastoma metastasis. Several small areas of signal loss were observed in the kidneys of chick embryos. Arrows indicate the areas where signal loss with T_2_*W FLASH was maintained or increased (for quantification of the signal loss, see supplemental Figure 1), indicating the presence of metastasizing labeled cells. Organ dissection and analysis by fluorescence microscopy confirmed the presence of several metastatic deposits in the kidney as shown in Figure 3D. The metastatic deposits consisted of up to 12 cells and up to 4 MPIOs. Thus, even very small metastasis could be detected by MRI. However, their identification was not trivial given their small size in the inherent low MRI signal of the kidney. An exact registration between MRI and histology was not possible as the MRI data acquired were nonisotropic, therefore anatomical landmarks were used and provided a good correlation between imaging findings and histological staining.

To be able to differentiate the small metastatic deposits from the blood vessels, we applied ToF MRA. This allowed signal loss caused by small blood vessels to be distinguished from potential metastasis more effectively than using FLASH alone. As ToF is dependent on the influx of fresh unsaturated blood, chick movement reduction was necessary. We tested 2 methods for reducing embryo movement: ketamine anaesthesia and embryo cooling. Cooling the embryos resulted in a reduced blood flow making successful ToF acquisition unfeasible. Therefore, ketamine anaesthesia was used. Representative ToF images were overlaid with imaged from T_2_W and T_2_*W FLASH scans obtained at E14 are shown in Figure 3E. Although ToF MRA allows the detection of bigger blood vessels, the small and very fine vessels in the kidney for example as well as other hypointense areas such as the gastrointestinal tract could not be resolved with the current acquisition protocol that was optimized to keep the embryos viable limiting the ability to detect small metastasis in this model.

Taken together, we have demonstrated that primary tumor formation on the CAM can be easily detected in the chick embryo model. Tumor cells within the organs of the chick embryo can be also detected, however 12 cells (labeled with 1 remaining MPIO) seemed to constitute the lower limit for a reliable detection and we anticipate that larger metastases are required to provide a more robust signal.

## Discussion

Much of our current understanding about the complex metastatic process comes from modern imaging techniques. Although each imaging modality comes with advantages and limitations, MRI offers detailed 3-D anatomical information and high resolution over time in a noninvasive manner. In agreement with others, we show here, that MRI is a powerful imaging modality for the study of tumor progression^17,21^ and embryonic development^16,22-26^ in the chick embryo. Compared to optical imaging, it can be used to detect the presence of tumors even when they are hidden beneath the egg shell and allows the noninvasive study of tumor progression and volume over time.

The main aim of this study was to determine whether MRI can also be used to detect the presence of metastasis noninvasively in the chick embryo. In order to observe metastatic dissemination, neuroblastoma cells were labeled with MPIOs as contrast agents. The labeling of cancer cells with MPIOs did not alter tumor formation on the CAM. While it did not offer significant advantages for primary tumor detection compared to unlabeled cells, it was necessary for small metastasis detection in the chick embryo organs. We initially tried to detect large clusters of cells, administered directly to the brain of the chick embryo, which resulted in a substantial loss of signal and thus in a robust detection of cancer cells. This suggests that cancer lesions of about 2 mm are detectable in the chick embryo, a size that is smaller than the MRI detection limit of metastasis reported being 10 to 20 mm in rodents.^27^ This finding is in agreement with others that have used superparamagnetic iron oxide nanoparticles (SPIONs) to successfully detect micrometastases in lung, lymph node, and brain in mice.^3,14,28^ Using SPIONs and hyperpolarized ^3^He MRI, Branca et al could detect micrometastasis of 0.3 mm in the lung of mice,^28^ while Foster et al could detect 100 MPIO-labelled cells by MRI after injecting them directly into the lymph node of mice^3^ and Heyn et al used SPION-labeled breast cancer cells to detect a small number of cells in the brain of mice.^14^ We have also shown previously that MRI can be used to reliably detect cell clusters of 5 × 10^4^ SPION-labeled cells in the brain *ex vivo*.^6^ Apart from SPIONs, other contrast agents have also been used for imaging metastasis. In mice, Zhou et al. could detect breast cancer metastases of less than 0.5 mm in different organs such as the lung, liver, lymph node, adrenal gland, and bone using a gadolinium-based contrast agent.^29^ Xue et al. developed a protein-based contrast agent that enabled them to image early liver metastases as small as 0.24 mm in diameter after tail vein injection of uveal melanoma cells into mice.^27^ It should be noted that although MPIO-labeling aids the detection of metastasized cells by enhancing their contrast, the division of cancer cells will lead to an expected loss of signal. The use of micron-sized contrast agents, as used here, offer the advantage that at least one of the daughter cells could potentially retain enough iron to display a T2-shortening effect, which would be lost in the case of nano-sized particles, where a 50% reduction in signal intensity at every cell division would quickly render them undetectable. However, this means that in a rapidly dividing cancer type, a proportion of unlabeled cells exists that thus will not be detectable.

Compared to mice, the chick embryo is a cost effective and convenient model, complying with the 3Rs by replacement of animal use. At E14, the metastatic deposits of neuroblastoma cells consist of only few cells, hence we wanted to determine if MRI could be used for their identification. We could observe signal reduction caused by a single MPIO particle and thus identify very small clusters of metastasized cells in the kidneys. However, the signal observed in the chick’s internal organs, including the kidney is inherently low, hence reliable detection of metastatic cells remains challenging with a potential of increased false positives. A confirmatory method, such as dissection, was needed to confirm the presence of such small metastatic deposits. These detection difficulties can be partially overcome by applying special techniques, such as ToF MRA, which we used here. Although ToF MRA enabled us to identify larger blood vessels, very small blood vessels couldn’t be resolved and consequently failed to facilitate the reliable detection of small metastasis in organs such as the kidney. Hence we would recommend ToF for cases where cells consistently metastasize to a defined region, which could then be scanned using a narrower field of view with a shorter scan time. Thus, the detection of metastasis of tumor types that disseminate either in organs of minimal signal loss, such as the brain or disseminate in bigger cell clusters, is more appropriate to this model.

In conclusion, we report that MRI is a suitable and highly sensitive imaging modality to image tumorigenesis *in ovo* using a chick embryo. We could, for the first time, identify metastatic deposits in the chick embryo by MRI. However, for reliable detection, we observed that 12 cells was the lower limit of detection. While this means that this approach cannot be used to detect the onset of metastasis from a single cell, the small metastases observed was still remarkable, with the potential of providing longitudinal view of disease progression in the same animal noninvasively, particularly of primary tumors generated in areas such as the CAM or injected cells in the brain.

## Supplemental Material

supplement_figure - Magnetic Resonance Imaging for Characterization of a Chick Embryo Model of Cancer Cell Metastases Click here for additional data file. supplement_figure for Magnetic Resonance Imaging for Characterization of a Chick Embryo Model of Cancer Cell Metastases by Anne Herrmann, Arthur Taylor, Patricia Murray, Harish Poptani, and Violaine Sée in Molecular Imaging
