# Supplementary material for: Magnetic Resonance Imaging for Characterization of a Chick Embryo Model of Cancer Cell Metastases
Source: Mol Imaging. 2018 Nov 5;17:1536012118809585. doi: 10.1177/1536012118809585 (PMC6236852; doi:10.1177/1536012118809585)
Supplement: supplement_figure - Magnetic Resonance Imaging for Characterization of a Chick Embryo Model of Cancer Cell Metastases [file supplement_figure.pdf]

A

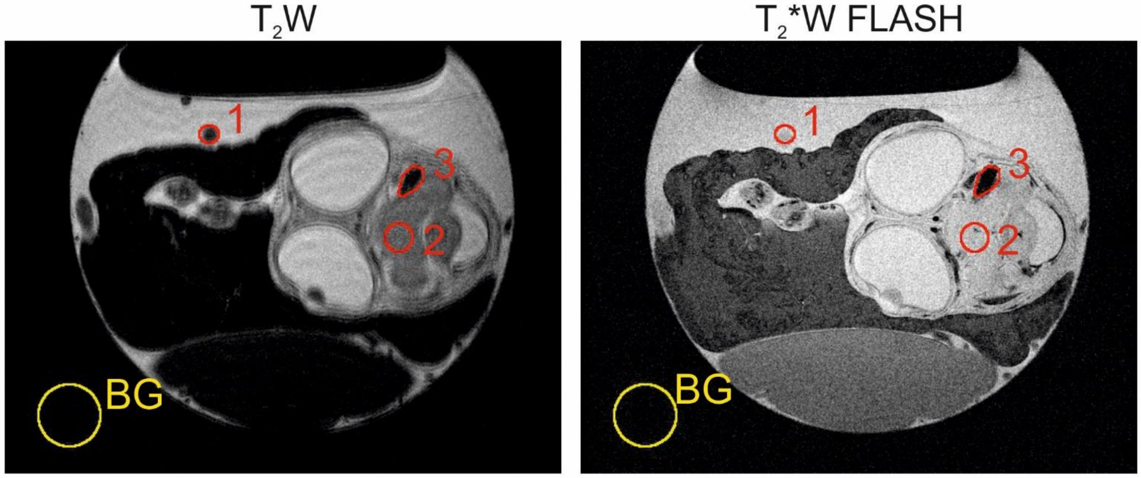

B

		T_2W			T_2*W FLASH		
		Signal	SNR	CNR	Signal	SNR	CNR
S_{BG}		13.89			23.49		
δ_{BG}		78.23			13.04		
S_{ROI}	1	95.01	10.74		188.65	52.24	
	2	104.24	11.79	0.06	185.54	51.38	0.07
	3	53.60	6.06	0.11	50.06	13.86	0.28

Supplemental Figure 1

A) Representative sagittal T_2W and T_2*W FLASH MR images of E14 chick embryo *in ovo* as shown in Figure 3A. Regions of interest (ROI) are indicated as follows: Background (BG) = yellow circle, ROI 1 = extraembryonic blood vessel, labelled with red circle, ROI2 = part of the chick embryo brain, labelled with red circle, ROI3 = area containing MPIO labelled cells located within the brain of the chick embryo surrounded by red outline. **B)** Table of signal intensity of background (SBG) as well as the 3 ROIs displayed in A, standard deviation of the background (δBG), signal to noise ratio (SNR) and contrast to noise ratio (CNR). Signal intensity (mean grey value) was measured in the ROI. SNR and CNR were calculated using following formulas SNR = SROI/sqrt(δBG), CNR = (SROI3/SROI2 or SROI3)/SQRT(δBG).
